# 5′/ 3′ imbalance strategy to detect ALK fusion genes in circulating tumor RNA from patients with non-small cell lung cancer

**DOI:** 10.1186/s13046-018-0735-1

**Published:** 2018-03-27

**Authors:** Yongqing Tong, Zhijun Zhao, Bei Liu, Anyu Bao, Hongyun Zheng, Jian Gu, Mary McGrath, Ying Xia, Bihua Tan, Chunhua Song, Yan Li

**Affiliations:** 1 Department of Clinical Laboratory, Renmin Hospital of Wuhan University, 99 Ziyang Road of Wuchang District, Wuhan, 430060 People’s Republic of China; 2 Laboratory Medicine Center of General Hospital of Ningxia Medical University, Yinchuan, 750004 People’s Republic of China; 3 Department of Pathology Affiliated Tianyou Hospital of Wuhan University of Science and Technology, Wuhan, 430064 People’s Republic of China; 4 Pennsylvania State University College of Medicine and Hershey Medical center, Penn State Hershey Children’s Hospital, PO Box 850, 500 University Drive, Hershey, PA 17033 USA; 5 Department of Hematology, The First Affiliated Hospital of Anhui Medical University, Hefei, Anhui 230022 People’s Republic of China

**Keywords:** Non-small cell lung cancer, Circulating tumor RNA, Fusion gene, ALK, 5′/3′ imbalance

## Abstract

**Background:**

Detecting an ALK fusion gene in patients with non-small cell lung cancer (NSCLC) could provide evidence to guide individualized therapy.

**Methods:**

The 5′/3′ imbalance strategy for quantitative reverse transcription-PCR (RT-qPCR) was developed to detect ALK fusion genes in circulating tumor RNA (ctRNA) of NSCLC patients.

**Results:**

This method was validated in patients with the ALK fusion gene confirmed by next generation sequencing (NGS). The amount of the ALK fusion gene detected by the new method ranged from 33.2 to 987.4, (mean 315.2), in the patients confirmed to have the ALK fusion gene (+). This is much higher than the amount of fusion gene detected in the patients who are negative for the ALK fusion gene (−). The amount detected in the ALK fusion gene (−) samples ranged from 0.36 to 13.04, (mean 4.58). In 188 NSCLC patients, the specificity and sensitivity of the method was compared to that of the FISH method. About 10.64% of the patients showed higher ALK fusion gene expression, and were classified as ALK fusion gene (+). This is identical to the percentage of patients detected by the FISH method to be ALK fusion gene (+). The cutoff value for diagnosis of ALK fusion (+) is 32.9 as determined by this method.

**Conclusions:**

A new RT-PCR method using a 5′/3′ imbalance strategy was developed, with high specificity and sensitivity, for detection of the ALK fusion gene in ctRNA of NSCLC patients. This method can rapidly detect ALK fusion genes in patients, which will be helpful for guiding targeted therapy, particularly the individualized usage of TKIs in these patients.

## Background

Non-small cell lung cancer (NSCLC) is the most common human malignancy with the highest morbidity and mortality [1]. With the progression of NSCLC therapies, and clinical application of targeted therapies such as the tyrosine kinase inhibitors (TKIs), crizotinib and gefitinib, the survival of patients has significantly improved [2]. Crizotinib is a small molecular compound targeting tyrosine kinase in the anaplastic lymphoma kinase (ALK) fusion gene. These compounds mainly benefit the highly malignant NSCLC patients who have a positive ALK fusion gene [3, 4].

ALK fusion genes are present in 3 to 7% of patients with NSCLC [5]. A variety of ALK fusion genes such as EML4-ALK, KIF5B-ALK, KLC1-ALK, TFG-ALK, TPR-ALK, HIP1-ALK, STRN-ALK, DCTN1-ALK, SQSTM1-ALK, BIRC6-ALK and BCL11A-ALK have been reported in NSCLC patients [6–9]. The current methods for detecting ALK fusion genes include chromosome karyotyping, FISH, RT-qPCR and NGS [10]. Owing to the diversity and complexity of the fusion genes, traditional FISH and RT-qPCR methods are greatly limited in clinical application [10]. Particularly, the current methods for detecting ALK fusion genes require tumor tissue specimens, such as surgically resected or biopsied tumor tissue, or malignant pleural effusion fluid [11]. However, it is often difficult to obtain tumor tissue in the NSCLC patients, especially in advanced phases after radiotherapy or chemotherapy. Studies have shown that a large amount of free RNA exists in the peripheral blood of the normal population [12, 13]. Circulating tumor RNAs (ctRNA) also exists in the peripheral blood of cancer patients such as those with melanoma [14, 15] and breast cancer [16]. These ctRNA may also be used as an effective specimen for diagnosis in tumors [17–19].

**Fig. 1:**
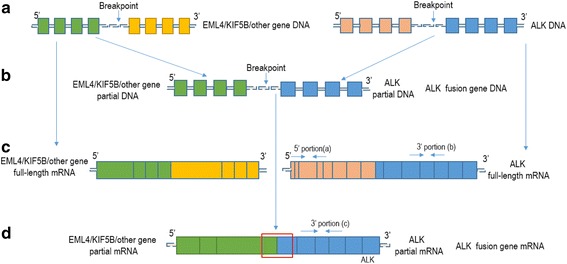
Strategy for the detection of imbalanced expression of the ALK fusion gene 5′/3′ portion. **a** shows ALK and the partners’ genomic DNA structures and breakpoint locations. **b** shows the ALK fusion gene in which the 3′ portion of the ALK gene (right) is fused with the 5′ portion of the partners (left). **c** shows the normal mRNA structure of the ALK gene (right) and the partners (left). The a or b expression level represents the ALK gene (a = b). **d** shows the ALK fusion gene mRNA structure, in which the 3′ portion of the ALK gene (right) is fused with the 5′ portion of the partners (left). The c expression level represents the ALK fusion gene

The ALK gene, which has 29 exons, usually has a breakpoint between exon 19 and exon 20 in NSCLC [20] (Fig. 1a). The 3′ portion can be maintained and the 5′ portion can be lost when the breakpoint happens (Fig. 1b). Therefore, analysis of ALK gene expression after exon 20 could represent the expression level of either the entire ALK genes or the fusion gene. The expression of the ALK gene on the left and right sides of the breakpoint was detected by RT-qPCR with two pairs of ALK primers respectively (Fig. 1c and d). The PCR product on the left side of the breakpoint represents the 5′ portion (a) of the ALK gene, and the right side represents the 3′ portion (b) of the ALK gene (Fig. 1c). In the presence of the ALK fusion gene, the 5′ portion (a) of the ALK gene is lost but the 3′ end remains (c) (Fig. 1d). The expression of the ALK gene at the 3′ portion will increase, and the ratio between the expressions of the 3′ portion (b + c) versus the 5′ portion (a) (3′/ 5′) should be increased in the presence of the ALK fusion gene (Fig. 1c and d). This strategy could effectively detect the ALK fusion gene no matter which partner genes were at the 5′ portion of the fusion genes. In short, the c portion does not exist if there are no ALK fusion genes, then b + c/ a = 1; and the c portion exists if there are ALK fusion genes, then b + c/ a˃1 (a = b). Therefore, our study intends to use an imbalance strategy of 3′ and 5′ portion expression of ALK genes to detect ALK fusion genes in ctRNA from NSCLC patients.

The purpose of this study is to establish a rapid detection method for ALK fusion genes in plasma ctRNA from patients with NSCLC by the imbalance strategy, i.e. measuring the difference of 5′/ 3′ portion expression of the ALK gene. This study will be helpful for clinical diagnosis and determination of need for targeted therapy with TKIs in NSCLC patients.

## Methods

### Patients and objectives

Sixty-six inpatients with NSCLC were evaluated for ALK fusion genes expression by NGS (Thermo Fisher, USA) from January 2016 to March 2017 in the Department of Oncology of Renmin Hospital of Wuhan University. 31 patients (average age: 54.9 ± 17.5 years old) were AKL fusion gene positive, and 35 patients (average age: 47.6 ± 18.1 years old) were ALK fusion gene negative. Also, 30 healthy controls (average age: 41.5 ± 16.4 years old) were randomly selected from the Physical Examination Center, and the 30 patients (average age: 43.7 ± 13.1 years old) with pneumonia were from the Department of Respiratory Medicine, Renmin Hospital of Wuhan University. Plasma samples and paraffin specimens from 188 NSCLC patients, who were confirmed to be negative for the EGFR, KRAS and BRAF genes, were selected in this study for screening of the ALK fusion genes with the new RT-qPCR method and FISH. All the patients provided their written informed consent in accordance with the Declaration of Helsinki before enrollment in the study. The study was approved by the Institutional Review Board of Renmin Hospital of Wuhan University.

### Sample collection and treatment

5 ml EDTA- anticoagulated peripheral blood was obtained from the individuals and the plasma was prepared. The plasma RNA was isolated with the plasma cell-free RNA isolation kit (Qiagen, No. 73404) in 2 h. In this kit, the DNase enzyme is used to digest the possibly contaminated genomic DNA. The ratio of OD260 / OD280 was measured. The RNA concentration per sample was 0.13 to 89.7 ng/ml. 2 μl of the total plasma RNA for each patient was used for reverse transcription to cDNA with the reverse transcription kit (Dalian Biotech Biotechnology, Cat. No. 6210A), and 2 μl of cDNA template was used for qPCR.

### The expression of the 5′/3′ portion of the ALK gene detected by RT-qPCR

The primers and probes for exon 20 (E20) and exon 3 (E3) of the ALK gene, and exon 3 (E3) of the ACTB gene (used as the internal reference), were designed with Primer Express 3.01 (ABI, USA) (Table 1). The forward primer for exon 20 and exon 3 covered nucleotides from exon 19 and exon 2 respectively. The paired primers can only detect the mRNA, not the genomic DNA. The total volume of the RT-qPCR reaction system consisted of 30 μl as follows: 1 μl of 5pM for each primer (forward and reverse primers), 1 μl of 2.5pM probes for E20 and E3 of the ALK gene, and E3 of the ACTB gene, 15 μl of PCR mix, 2 μl of cDNA template, and 4 μl of deionized water. Reaction conditions were: 94 °C for 10 min; 94 °C for 30 s, 62 °C for 45 s, for a total of 45 cycles, and 62 °C for collection of fluorescent signals.

**Table 1:** The primer and probe for detecting the 3′/5′ portion of ALK gene with RT-qPCR

Gene	Exon	Primer(5′ → 3′)	Located on cDNA	Amplicon length
ALK	E20	Forward: CGGCATCATGATTGTGTACC	3159~ 3178	145 bp
		Reverse: CTTGCCAGCAAAGCAGTAG	3285~ 3303	
		Probe: FAM-CTGAGCAAGCTCC-MGB	3238~ 3303	
	E3	Forward: CAGCCGATATGGTCTGGAG	777~ 795	168 bp
		Reverse: ATCTCCTTAGAACGCTCTGC	925~ 944	
		Probe: VIC-TCCCCTCCACTGCAT-MGB	829–843	
ACTB	E3	Forward: CAGGCACCAGGGCGTGAT	114~ 131	134 bp
		Reverse: CCATGTCGTCCCAGTTGGT	229~ 247	
		Probe: NED-ACGAGGCCCAGAGCA-MGB	167~ 181	

### Calculations of the expression of ALK fusion genes

The expression levels of ALK E20 and E3 were detected by the LightCycler 480 fluorescence spectrometer, and the expression of ALK E20 and E3 was calculated by 2^-△CT^ with the formula 2^-△CT^ = 2^-[CT(ALK E20)/(ALK E3)-CT(ACTB)]^. The expression of the ALK fusion gene was calculated by 2^-△△CT^ with the formula 2^-△△CT^ = 2^-[(CT(ALK E20)- CT(ACTB))-(CT(ALK E3)-CT(ACTB))]^.

### Fish

FISH was used to detect ALK rearrangements in formalin-fixed and paraffin-embedded specimens using a commercially available break apart probe for the ALK gene (Vysis LSI ALK Dual Color; Abbott Molecular) in accordance with the manufacturer’s instructions. FISH was carried out on the same tissue area. The probe hybridizes to band 2p23 on either side of the ALK gene breakpoint. The 5’ ALK signal was labeled with Spectrum Green (green), and 3’ ALK signal with Spectrum Orange (orange). Criteria for the probe signal interpretation in at least 100 interphase nuclei were as follows: (i) separated green and orange signals, or single red signal identified in ≥15% (≥15/100) of tumor cells analyzed indicated rearranged ALK; (ii) overlapping signals, adjacent signals, or overlap of red and green signals (yellowish) less than 2 signal diameters apart indicated cells in which ALK was not rearranged.

### Statistical analysis

The data were statistically analyzed using GraphPad Prism 7.0 software. The expression of the gene was expressed as the mean ± SD; and the difference between the two groups was statistically analyzed by a student T-test. A *P* value less than 0.05 (*P* < 0.05) was considered to be significant.

## Results

### ALK expression by detection of the 5′ portion or the 3′ portion with RT-qPCR

**Fig. 2:**
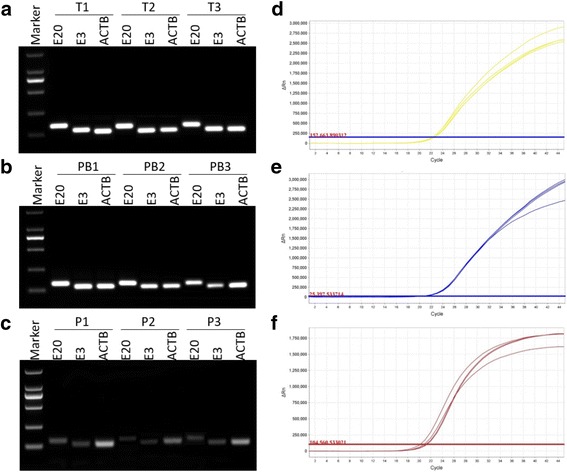
Evaluation of the primers’ specificity for detection of the expression of the 3′/5′ portion of the ALK gene. (**a**-**c**) RT-PCR products of the 3′/5′ portion of the ALK gene from tumor tissues (**a**), PBMCs (**b**) and plasma (**c**) are shown on agarose gel. **d** and **e** show the expression of the 3′/5′ portion of the ALK gene, respectively, by RT-qPCR. **f** shows the expression of ACTB by RT-qPCR as a control. T = tumor tissue, PB = peripheral blood, P = plasma, E3: ALK gene exon 3; E20: ALK gene exon 20. ACTB: beta-actin

Total RNA from NSCLC tumor tissues, patients’ plasma, and healthy donor peripheral blood mononuclear cells (PBMCs) were extracted, and cDNA was generated. The 5′/3′ portion of the ALK gene was amplified by RT-PCR with specific primers on E20 or E3 respectively (Fig. 1). The PCR products from 3 different tumor tissues (T1-T3), PBMCs (PB1-PB3) and plasma (P1-P3) are shown on agarose gels (Fig. 2a-c). These results indicate the designed primers could effectively amplify PCR products with expected size from the RNA isolated from tumor tissues, patients’ plasma and PBMCs. The RT-qPCR data also showed that the primers could efficiently amplify the 5′ or 3′ portion of the ALK gene (Fig. 2d-f), and ALK expression could be determined by these methods.

### Detection of the ALK fusion gene by measuring the ratio of the 3′ portion versus the 5′ portion of the ALK gene

**Fig. 3:**
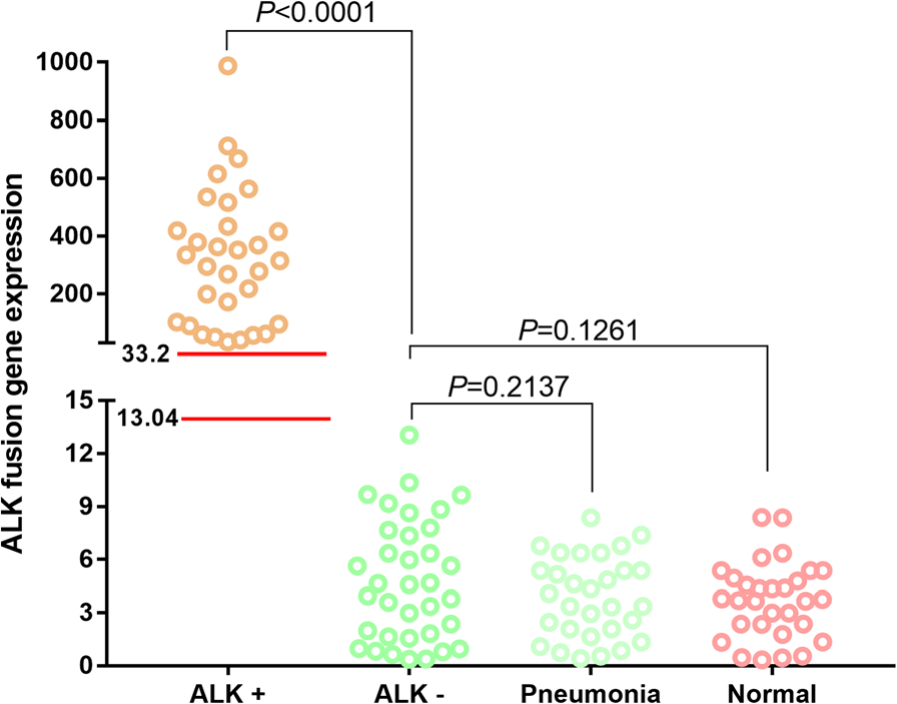
Expression of the ALK fusion gene in the plasma of ALK fusion gene (+ / -) NSCLC patients. ALK+ showed the positive expression of ALK fusion in the tumor tissue in NSCLC patients. ALK- showed the negative expression of ALK fusion in the tumor tissue with NSCLC patients. Pneumonia showed the patients with pulmonary infection as their clinical diagnosis. Normal showed the healthy people as a control. The ALK fusion gene expression was confirmed though NGS

The expression of the ALK fusion gene was detected in 66 NSCLC patients who were either ALK fusion gene (+) (31 cases) or (−) (35 cases) using RT-qPCR for both the 5′ and 3′ portion. The ratio of the 3′ portion versus 5′ portion of the ALK gene was further calculated for each patient, which represents the expression level of the ALK fusion gene. The expression of the ALK fusion gene was 33.2 to 987.4 (mean 315.2) in plasma of ALK-positive NSCLC patients, and 0.36 to 13.04 (mean 4.58) in that of ALK-negative NSCLC patients (Fig. 3). The lowest value of fusion expression was 33.2 in the ALK fusion gene (+) group, which was significantly higher than that of the highest expression in the ALK fusion gene (−) group (13.04) (Fig. 3). The expression levels of ALK fusion genes in healthy controls and patients with pneumonia were 0.34 to 8.37 (mean 3.72) and 0.41 to 8.35 (mean 3.72) respectively (Fig. 3). Statistical analysis showed that expression of the ALK fusion gene is significantly higher in the plasma of patients who were ALK fusion gene (+) than those who were fusion gene (−), and also higher than expression in the healthy and pneumonia patients (*P* < 0.01). There is no significant difference in expression between the ALK fusion gene (−), normal, and pneumonia patients (*P* > 0.05) (Fig. 3). These data show the results of ALK fusion gene expression are similar between the RT-qPCR method and NGS.

### Screening of ALK rearrangements by RT-qPCR and FISH in clinical NSCLC samples

**Fig. 4:**
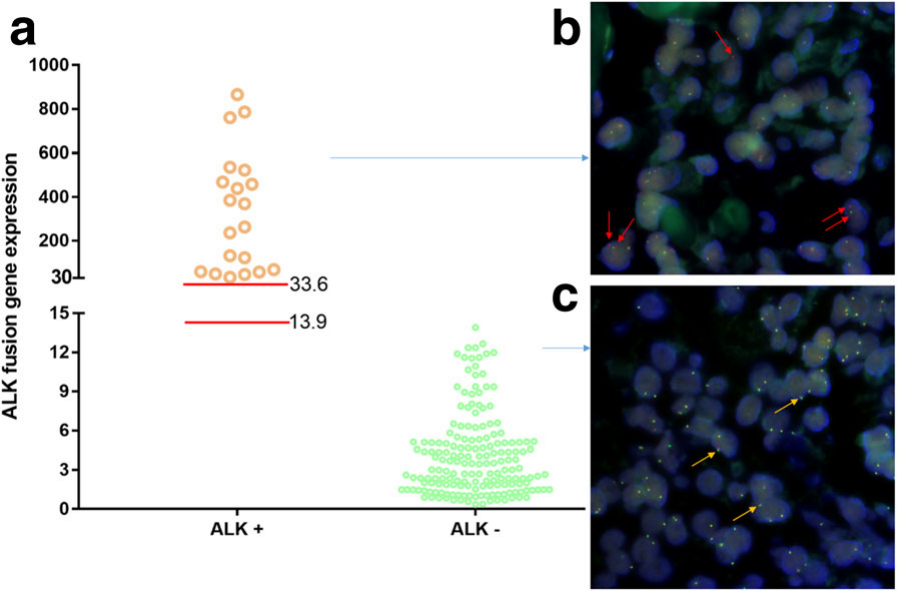
Analysis of ALK fusion genes in 188 plasma samples of NSCLC patients by RT-qPCR and FISH. (**a**) ALK fusion genes are determined by 3′/5′ portion RT-qPCR of the ALK gene. The expression of ALK gene was calculated using 2^-△△CT^ method. (**b**-**c**) The representative FISH images for patients with ALK fusion gene (+) (**b**) and ALK fusion gene (−) (**c**). Red arrow, split red-green dots or single red dots are indicative of ALK rearrangement; yellow arrow, or touching red-green dots are indicative of no ALK rearrangement

The 5′/3′ portion RT-qPCR was used to detect the expression of ALK rearrangements in plasma ctRNA from 188 patients with NSCLC, and FISH was used to detect ALK rearrangements in the corresponding paraffin specimens. The RT-qPCR data showed that ALK expression could be divided into two groups: one is higher than 33.6 (33.6 to 865.4 (mean 316.1), the other one is lower than 13.9 (0.365 to 13.9 (mean 3.3) (Fig. 4a). The 20 cases of patients with higher ALK expression (> 33.6) are the ALK fusion (+) group, with a positive rate of 10.64% (20/188) (Table 2). The FISH results also show this group has ALK fusion genes (Fig. 4b and Table 2). The 168 cases of patients with lower ALK expression (< 13.9) are the ALK fusion gene (−) group, with a negative rate of 89.36% (168/188). The FISH results also show they are ALK fusion gene (−) (Fig. 4c).

**Table 2:** Correlation of the expression of ALK fusion gene detected by RT-qPCR and FISH

Cases	RT-qPCR^a^		FISH		Pathol.
	expression value	+/−	+/−	%	
021	33.6	+	+	18	A
124	439.1	+	+	68	AS
065	123	+	+	38	A
075	263	+	+	53	AS
184	58.9	+	+	23	A
046	49	+	+	20	A
089	67.9	+	+	27	A
102	759.9	+	+	90	A
018	132.35	+	+	41	A
068	236.5	+	+	49	A
135	468.3	+	+	73	A
006	523.1	+	+	77	A
029	536.2	+	+	79	A
173	458.2	+	+	70	AS
084	369.1	+	+	61	A
003	786.2	+	+	92	A
044	865.4	+	+	93	A
148	385.2	+	+	63	A
039	46.2	+	+	20	A
017	56.8	+	+	23	A

NOTE: +, positive; −, negative; Pathol., pathology; A, adenocarcinoma; AS, adenosquamous carcinoma; ^a^Expression in 3′ portion of ALK mRNA relative to 5′ portion and expression value = 2^-△△CT^ = 2^-[(CT(ALK E20)- CT(ACTB))-(CT(ALK E3)-CT(ACTB))]^

**Fig. 5:**
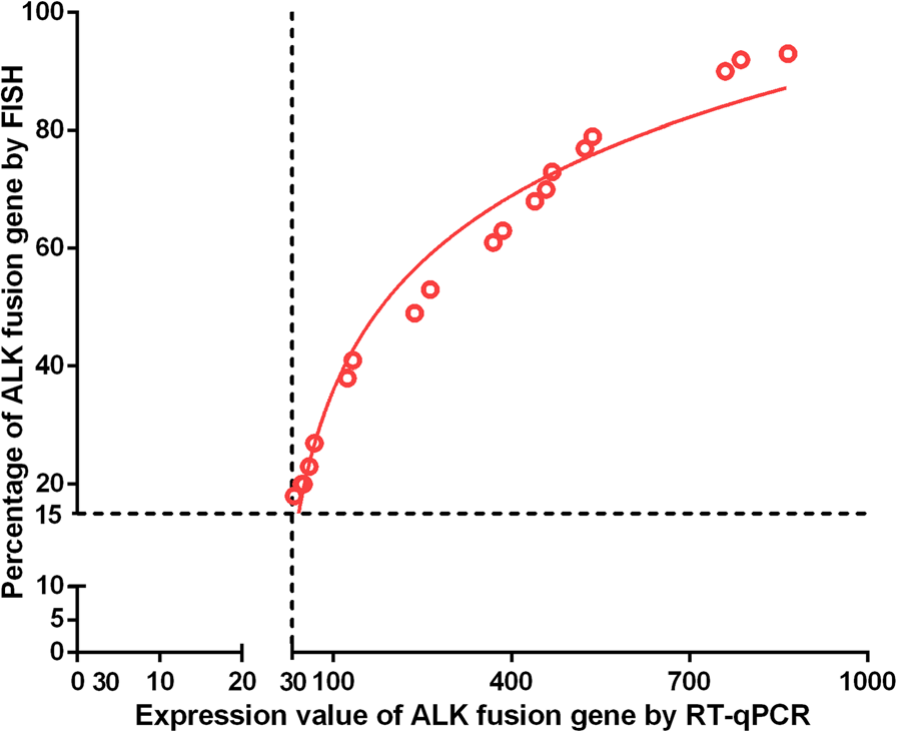
Correlation analysis of the expression value of the ALK fusion genes by 3′/5′ portion qPCR and the percentage of the ALK fusion (+) cells by FISH. The expression value of the ALK gene in the patients with ALK fusion genes has a significant positive correlation with the percentage of the ALK fusion (+) cells identified by FISH in the patients. The lowest cross point indicated the expression value 32.9 on the X axis corresponding to that of 15% ALK fusion (+) cells on the Y axis by the FISH method

Both the RT-qPCR and FISH method detected the ALK fusion gene (+) in 20 cases. Three of these cases with the ALK fusion gene (+) are adenosquamous carcinoma, and the remaining 17 cases are adenocarcinoma (Table 2). If the fusion gene (+) cells are < 15% in the patient sample, the patient is considered to be fusion gene (−) by FISH analysis. Our data showed that the ALK fusion gene (+) cells when detected by FISH had 18% to 93% fusion gene (+) cells in the patient samples. We also analyzed the correlation of the percentage of ALK fusion gene (+) cells detected by FISH with ALK fusion gene expression detected by qPCR, and found they are highly consistent (Table 2) and have a significant positive correlation (Fig. 5).

These data indicate that the 5′/3′ portion RT-qPCR data is consistent with that of the FISH results. It also demonstrates that the specificity of the 3′/5′ portion qPCR method is comparable to that of the FISH method. From Fig. 5, we also show that the expression value of the ALK gene is 32.9 by the RT-qPCR method and the percentage of the ALK fusion (+) cells in the patient sample is 15% by FISH. This data indicates that the cutoff value is 32.9 for this method of diagnosis of ALK fusion (+) in the patient plasma samples.

## Discussion

The main purpose of this study is to detect the expression of the ALK fusion gene by 5′ and 3′ end portion RT-qPCR, to evaluate the significance of ALK fusion gene detection in ctRNA from NSCLC patients, and to further provide the molecular basis for targeted therapy in NSCLC patients. The frequency of ALK fusion genes is only about 3% to 7% in NSCLC patients [21]. Data indicates that TKIs such as crizotinib or ceritinib are not useful for more than 90% of NSCLC patients [22]. It is recommended that these chemotherapies only be used for patients who are ALK fusion gene (+) [3]. Therefore, the presence or absence of the ALK fusion gene is critical in determining therapy options for patients [23, 24].

There are at least 12 kinds of ALK fusion genes such as EML4-ALK, KIF5B-ALK, KLC1-ALK and TFG-ALK in NSCLC patients [6, 25–27]. The traditional RT-qPCR assay for detection of ALK fusion genes is with forward primers and reverse primers which are designed on two different genes in the fusion gene [28, 29]. Thus, a pair of primers could only detect one subtype of a specific fusion gene. Currently we lack a method that can detect different subtypes of the same fusion gene and also detect unknown new subtypes by the same pair of primers.

We used the 5′ and 3′ expression imbalance strategy to detect the expression of the ALK fusion genes through the ratio of RT-qPCR values for the 3′ portion versus the 5′ portion of the ALK gene in the ctRNA from patient samples. This method cannot determine which partner genes are fused with the ALK gene, or the subtypes of the ALK fusion genes. However, this method can detect all types of ALK fusion genes. More specifically, any existing ALK fusion gene in patient samples can be detected no matter what types of ALK fusion genes are present, including unidentified new ALK fusion genes (Fig. 1d). Moreover, usually the amount of ctRNA in plasma is low and varies by patient. In this method, the ratio of the 3′ / 5′ portion of the ALK gene is used as the ALK fusion gene expression. The ratio is not changed no matter how much total RNA is used in the system. Therefore, this method is more accurate for detection of ALK fusion genes in ctRNA samples than other RT-PCR methods.

We also used the 5′ and 3′ expression imbalance strategy to detect ALK fusion genes in the plasma of ALK fusion gene (+) samples that were confirmed by NGS of tumor tissues. The ALK fusion gene expression in the plasma of ALK fusion gene (+) NSCLC patients was 33.2 to 987.4 (mean 315.2), which was significantly higher than that of ALK fusion gene negative NSCLC patients [0.36 to 13.04 (mean 4.58)]. There is no overlap between the two groups. This method could effectively distinguish the ALK fusion gene (+) and (−) samples (Fig. 3). This result is consistent with results from NGS, and also suggests that the RT-qPCR method with imbalance strategy is reliable for detection of fusion genes in ctRNA from NSCLC patients. Wang et al. [21] also reported that expression of ALK fusion genes from tumor tissue detected by a similar RT-qPCR method is consistent with that of other methods such as FISH, NGS, and chromosome karyotyping in NSCLC patients. Our data is consistent with these reports, and also further confirms the reliability and specificity of our RT-qPCR method on detection of the ALK fusion genes in ctRNA from patients with NSCLC.

Our data also showed 100% consistency with FISH detection of the ALK fusion gene positive and negative samples. It is well known that the gold standard for detecting ALK fusion gene (−), is if the fusion gene (+) cells are less than 15% in a patient sample by FISH. Our method showed the lowest value of ALK fusion gene expression (cutoff value), was 32.9 in ALK fusion gene (+) samples, which is significantly higher than the highest expression of 13.9 seen in ALK fusion gene negative samples. Our data also suggest that our RT-qPCR method with 5′ and 3′ imbalance strategy to detect the ALK fusion genes in the ctRNA samples from NSCLC patients may be more sensitive and accurate than that of the FISH method (Fig. 4).

## Conclusions

We developed a new RT-qPCR method with 5′ and 3′ imbalance strategy to detect ALK fusion genes in the ctRNA from NSCLC patients. This method can effectively overcome the problem of lacking tumor tissue. Particularly it is a very valuable method for monitoring efficacy of, and resistance to chemotherapy drugs in the process of treatment when tumor tissue from surgery is not available. This method is also less time consuming, allows for easy handling and requires minimal staff and instruments compared to NGS, FISH and other methods [30]. This method can be applied more easily to clinical practice.

## Abbreviations

ALK: Anaplastic lymphoma kinase
ctRNA: Circulating tumor RNAs
EGFR: Epidermal growth factor receptor
FISH: Fluorescence In Situ Hybridization
NGS: Next-Generation Sequencing
NSCLC: Non-small cell lung cancer
RT-qPCR: Quantitative reverse transcription PCR
TKIs: Tyrosine kinase inhibitors
